# Inflammatory and Metabolic Predictors of Mortality in Pulmonary Thromboembolism: A Focus on the Triglyceride–Glucose Index and Pan-Immune Inflammation Value

**DOI:** 10.3390/jcm13196008

**Published:** 2024-10-09

**Authors:** Murat Bilgin, Emre Akkaya, Recep Dokuyucu

**Affiliations:** 1 Department of Cardiology, Private Aktif International Hospital, Yalova 77720, Turkey; drbilginmurat61@gmail.com; 2 Department of Cardiology, Bossan Hospital, Gaziantep 27580, Turkey; dremreakkaya@hotmail.com; 3 Department of Physiology, Medical Specialization Training Center (TUSMER), Ankara 06230, Turkey

**Keywords:** pulmonary thromboembolism, pan-immune inflammation, triglyceride–glucose index, mortality

## Abstract

**Objectives**: We aimed to evaluate the importance of metabolic and inflammatory markers, specifically the Triglyceride–Glucose Index (TGI) and pan-immune inflammation value (PIV), in predicting mortality among patients diagnosed with pulmonary thromboembolism (PTE). **Materials and Methods**: A total of 450 patients diagnosed with PTE between December 2018 and December 2023 were included in his study. The diagnosis of PTE was confirmed by clinical presentation, laboratory tests, and imaging studies such as computed tomography pulmonary angiography (CTPA). Data were obtained from medical records, including demographic information, medical history, laboratory results, and clinical outcomes. **Results**: In terms of age, non-survivors were older on average (66.1 ± 11.8 years) compared to survivors (58.3 ± 12.4 years) (*p* = 0.03). In terms of gender, 55% of non-survivors and 45% of survivors were male (*p* = 0.111). Non-survivors had higher BMIs (28.3 ± 5.1) than survivors (25.7 ± 4.5) (*p* = 0.04). In terms of hypertension, 40% of non-survivors and 30% of survivors had hypertension (*p* = 0.041). In terms of diabetes, 35% of those who did not survive and 20% of those who survived had diabetes (*p* = 0.001). In terms of smoking, 25% of non-survivors and 15% of survivors smoke (*p* = 0.022). In terms of TGI, non-survivors had higher TGI values (12.1 ± 1.5) than survivors (5.9 ± 1.2) (*p* < 0.001). In terms of PIV, non-survivors had significantly higher PIV (878.2 ± 85.4) than survivors (254.5 ± 61.1) (*p* < 0.001). The risk factors found to be significantly associated with differentiation in the multiple logistic regression analysis included age, BMI, TGI, and PIV (*p* = 0.005, *p* = 0.002, *p* = 0.013, and 0.022, respectively). As a result, according to ROC analysis for patients who are non-survivors, age, BMI, TGI, and PIV were significant prognostic factors. The cut-off points for these values were >60, >27, >10, and >500, respectively. **Conclusions**: the TGI and PIV are strong markers for predicting mortality in PTE patients. The independent predictive value of age and BMI further demonstrates their role in risk stratification. We think that high TGI values and PIVs reflect underlying metabolic and inflammatory disorders that may contribute to worse outcomes in these patients.

## 1. Introduction

Pulmonary thromboembolism (PTE) is an important and potentially life-threatening cardiovascular condition that develops when fragments of thrombi, mostly occurring in the deep veins of the legs, block the pulmonary artery and/or its branches. Despite advances in diagnosis and treatment, PTE continues to be associated with high morbidity and mortality rates. Identifying prognostic markers and understanding their effects is crucial to improving patient outcomes [1,2,3,4].

Recent studies emphasize the importance of metabolic and inflammatory parameters in the prognosis of various cardiovascular diseases. Among these parameters, the Triglyceride–Glucose Index (TGI) has emerged as a reliable indicator of insulin resistance, a known risk factor for cardiovascular events. TGI calculated from fasting triglyceride and glucose levels has been proven to have the capacity to predict adverse outcomes in coronary artery disease (CAD) patients. However, the impact and role of TGI on PTE has not yet been adequately investigated [5,6,7,8].

Some studies in the literature indicate that the pan-immune inflammation value (PIV) can be used in the diagnosis and prognosis follow-up of metabolic and cardiovascular diseases [9,10,11,12,13,14,15,16]. The PIV, which has entered the literature in recent years, has been proposed as a value that comprehensively measures systemic inflammation created by combining various hematological parameters, including neutrophils, lymphocytes, monocytes, and platelets. Inflammation plays a critical role in thrombus formation, dissemination, and resolution in the pathophysiology of PTE. Therefore, the evaluation of the PIV together with the TGI may allow us to examine the inflammatory and metabolic status of PTE patients from a more holistic perspective and thus provide better prognostic information.

This study aims to examine the correlation between the TGI, PIV, and mortality rates in patients with PTE. By evaluating these factors, we aim to understand their combined influence on patient outcomes and assess their potential as prognostic markers. Insights into the relationship between metabolic dysfunction and inflammation in PTE may facilitate the development of targeted treatment strategies and enhance risk stratification in clinical practice.

## 2. Materials and Methods

### 2.1. Study Design and Study Population

This study was approved by the decision of the Ethics Committee of Health Sciences University. The Declaration of Helsinki protocol was followed in the research protocol (2024/65, 27 March 2024). The study was performed in accordance with the ethical standards as indicated in the 1964 Declaration of Helsinki and its later amendments or comparable ethical standards. Written informed consent was obtained from all participants using appropriate patient consent forms.

A total of 450 patients diagnosed with PTE between December 2018 and December 2023 were included in this study. The diagnosis of PTE was confirmed by clinical presentation, laboratory tests, and imaging studies such as computed tomography pulmonary angiography (CTPA). Patients with autoimmune diseases, end-stage malignancies, positive COVID-19 tests, and patients under the age of 18 were excluded from the study. All patients were initially assessed for the severity of their condition, and treatment was stratified based on risk. Intermediate- and high-risk patients received thrombolytic therapy, including systemic thrombolysis or catheter-directed thrombolysis, as indicated. Anticoagulation was provided as standard for all patients, with low-molecular-weight heparin (LMWH) being the first-line choice, followed by a transition to oral anticoagulants as per the ESC guidelines [17]. EKOS (Endovenous Laser Therapy) was selectively used in cases with significant thrombus burden. Regular follow-ups were performed to monitor response to therapy and adjust treatment accordingly. Data were obtained from medical records, including demographic information, medical history, laboratory results, and clinical outcomes. The PIV was computed by multiplying the SII value and the monocyte count. The Systemic Inflammatory Index (SII) was calculated by the total number of neutrophils × total number of platelets/total number of lymphocytes [12]. The Triglyceride–Glucose Index is calculated using the formula TGI = ln(Triglyceride [mg/dL] × Glucose [mg/dL]/2) [8].

### 2.2. Statistical Analysis

Statistical analyses were performed using SPSS software (version 27.0; IBM, Armonk, NY, USA). In the power analysis, when the effect size was taken as 0.30, the margin of error as 5%, and the power as 80%, it was concluded that each group should consist of at least 176 patients with a 1-degree of freedom chi-square test. Categorical variables were expressed as frequency and percentage, and continuous variables were expressed as mean ± standard deviation. The primary outcome was all-cause mortality. Comparisons between survivors and non-survivors were made using Student’s *t* test or the Mann–Whitney U test for continuous variables and the chi-square test for categorical variables. Logistic regression analysis was used to identify independent predictors of mortality and adjust for potential confounding factors. Variables with a *p* value < 0.05 in univariate analysis were included in the multivariate model.

## 3. Results

A comparison of laboratory and socio-demographic findings is shown in Table 1.

In terms of age, non-survivors were older on average (66.1 ± 11.8 years) compared to survivors (58.3 ± 12.4 years) (*p* = 0.032). In terms of gender, 55% of non-survivors and 45% of survivors were male (*p* = 0.111). Non-survivors had higher BMIs (28.3 ± 5.1) than survivors (25.7 ± 4.5) (*p* = 0.040). In terms of hypertension, 40% of non-survivors and 30% of survivors had hypertension (*p* = 0.041). In terms of diabetes, 35% of those who did not survive and 20% of those who survived had diabetes (*p* = 0.001). In terms of smoking, 25% of non-survivors and 15% of survivors smoke (*p* = 0.022). The percentage of patients who received systemic thrombolytic therapy was 35% (77 patients) in the non-survivor group and 50% (115 patients) in the survivor group (*p* = 0.01). Similarly, catheter-directed thrombolysis was administered to 15% (33 patients) of the non-survivors, compared to 25% (58 patients) in the survivor group (*p* = 0.03). Regarding EKOS, 5% (11 patients) of the non-survivors underwent the procedure, whereas it was performed in 12% (28 patients) of the survivors (*p* = 0.04). In the non-survivor group, 45% (99 patients) showed evidence of RV strain, and 20% (44 patients) had RV failure. Among the survivor group, 30% (69 patients) demonstrated RV strain, with RV failure observed in 10% (23 patients). In terms of TGI, non-survivors had higher TGI values (12.1 ± 1.5) than survivors (5.9 ± 1.2) (*p* < 0.001). In terms of the PIV, non-survivors had significantly higher PIVs (878.2 ± 85.4) than survivors (254.5 ± 61.1) (*p* < 0.001).

A univariate logistic regression analysis of factors used for non-survivors is shown in Table 2. The risk factors found to be significantly associated with non-survivors in the regression analysis included age, BMI, TGI, and PIV (*p* = 0.001, *p* = 0.007, *p* = 0.010, and 0.023, respectively) (Table 2).

Multiple logistic regression analysis of factors used for non-survivors is shown in Table 3. The risk factors found to be significantly associated with differentiation in the regression analysis included age, BMI, TGI, and PIV (*p* = 0.005, *p* = 0.002, *p* = 0.013, and 0.022, respectively) (Table 3).

**Figure 1:**
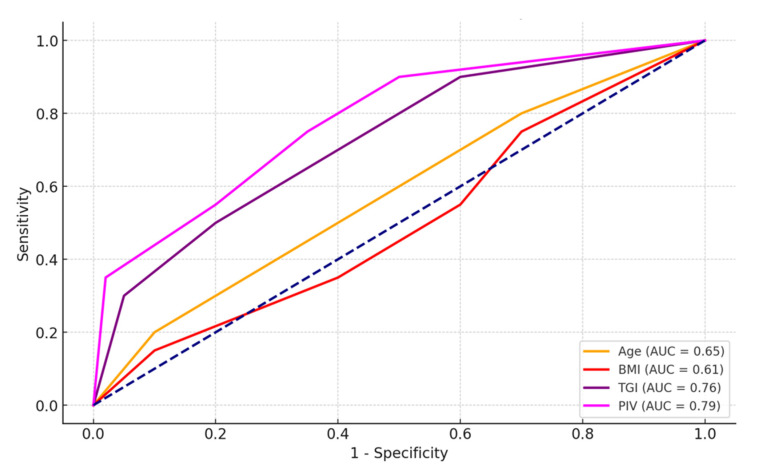
ROC analysis results in patients with non-survivors.

ROC analysis results in patients with non-survivors are shown in Table 4. ROC analysis results in patients with non-survivors are shown in Table 4. According to the results of ROC analysis in patients with non-survivors, a sensitivity of 60.0% and specificity of 60.0% for age (*p* = 0.002); sensitivity of 65.0%, and specificity of 55.0% for BMI (*p* = 0.031); sensitivity 78.0%, and specificity 73.0% for TGI (*p* < 0.001); sensitivity 82.0% and specificity 75.0% for PIV (*p* < 0.001). The cut-off points for these values were >60, >27, >10, and >500, respectively. As a result, according to ROC analysis for patients with non-survivors, age, BMI, TGI, and PIV were significant prognostic factors (Table 4, Figure 1).

## 4. Discussion

In our study, we show that the TGI and PIV are strong markers for predicting mortality in PTE patients. The independent predictive value of age and BMI further demonstrates their role in risk stratification. We think that high TGI values and PIV reflect underlying metabolic and inflammatory disorders that may contribute to worse outcomes in these patients. The results of our study are compatible with a limited number of studies in the literature.

In previous studies, the role of thrombolytic therapies, including systemic thrombolytics and catheter-directed thrombolysis, has been well documented in improving survival outcomes for patients with pulmonary embolism (PE) [18,19,20,21,22]. A study by Kearon et al. demonstrated that systemic thrombolytic therapy significantly reduced mortality rates in high-risk PE patients, supporting our finding that 50% of survivors received systemic thrombolytics compared to 35% of non-survivors (*p* = 0.01) [18]. In a study by Furfaro et al., it was stated that catheter-directed thrombolysis (CDT) reduces right heart strain and pulmonary artery pressures more quickly than anticoagulation, with low mortality rates (0–4%). While safer than systemic thrombolysis, CDT carries a slightly higher bleeding risk compared to anticoagulation alone [23]. In a study by Kabrhel et al., it was stated that CDT provides the highest quality-adjusted life years (QALYs) for patients, though it is costly and the difference in QALYs compared to anticoagulation is small. Systemic thrombolysis is associated with higher bleeding risks, while CDT shows promise with lower bleeding risks [22]. Similarly, CDT has been shown to enhance clot resolution and improve right ventricular function, as evidenced by Piazza et al., where catheter-directed therapy was associated with better survival outcomes [19]. Our results corroborate these findings, with 25% of survivors undergoing catheter-directed thrombolysis compared to only 15% of non-survivors (*p* = 0.03). Additionally, the use of EKOS, although less frequently studied, has been reported to offer benefits in carefully selected patients, particularly those with severe thrombus burden or contraindications to systemic thrombolytics by Tapson et al. [20]. Our data revealed a higher utilization of EKOS in survivors (12%) compared to non-survivors (5%), further indicating the potential efficacy of this modality (*p* = 0.04).

Several studies in the literature have demonstrated that the TGI is an important determinant of cardiovascular outcomes. In a study by Wang et al. (2024) involving patients with acute coronary syndrome (ACS), a higher TGI value was independently associated with an increased risk of major adverse cardiac events (MACE) during follow-up. The study suggested that the TGI may be a useful biomarker in ACS patients [24]. Cho et al. (2022) showed in a large cohort of Korean adults that a higher TGI value was associated with an increased risk of cardiovascular disease (CVD) and all-cause mortality. The study concluded that the TGI may serve as a simple and useful marker in identifying individuals at high risk of CVD and mortality [25]. Akbar et al. (2021) found that in patients with ACS, high TGI values were related to a higher risk of MACE during follow-up. The study highlighted that the TGI may be a useful marker for prognosis in ACS patients [26]. Yu et al. (2023) showed that the TGI was significantly related to carotid atherosclerosis independently of risk factors, suggesting that the TGI may be an early marker of atherosclerotic burden and cardiovascular risk [27]. In their study investigating the relationship between the TGI and long-term outcomes in patients with type 2 diabetes and CAD, Sun et al. (2024) stated that higher TGI levels are related to an increased risk of MACE and mortality. It has also been suggested that the TGI is a valuable prognostic marker in these patients [28]. Moon et al. (2023) investigated the association between TGI and CVD incidence in a large cohort of Korean adults. The study found that higher TGI levels were significantly associated with increased risks of CVD and all-cause mortality. It is concluded that the TGI may serve as a simple and effective marker in identifying individuals at high risk of CVD [29]. In our previous study, we assessed the predictive significance of clinical and laboratory parameters, including the THR, TGI, and PIV, in the differential diagnosis of ACS and their association with mortality. The results highlighted that those higher values of THR and the TGI, along with traditional risk factors such as age, cholesterol levels, diabetes, and smoking, were significantly linked with a higher likelihood of developing STEMI and increased mortality in ACS patients [30]. In the current study, we found that the TGI was significantly higher in survivors than in non-survivors. ROC curve analysis showed that the TGI had a good discriminatory ability in predicting mortality. Multiple logistic regression analysis confirmed that the TGI was an independent predictor of mortality in PTE patients.

Studies in the literature have revealed that the PIV is an important determinant of metabolic and cardiovascular outcomes. Xu et al. (2024) found that the PIV was a significant predictor of 28-day mortality in septic shock patients. Higher PIVs were related to increased mortality, highlighting the role of systemic inflammation in critical illness outcomes [31]. In a study of advanced non-small cell lung cancer (NSCLC) patients receiving immunotherapy, Chen et al. (2023) found that higher baseline PIVs were associated with worse progression-free survival (PFS) and overall survival (OS). It has been suggested that PIVs can be used to predict outcomes in cancer patients receiving immunotherapy [32]. In their study on patients with acute myocardial infarction (AMI), Goriki et al. (2020) revealed that in terms of the prognostic value of PIVs, higher PIVs were significantly associated with an increase in in-hospital stay and 6-month mortality. The study concluded that the PIV may serve as an effective predictor of adverse outcomes in patients with AMI, reflecting the underlying systemic inflammatory response [33]. In Murat et al.’s (2023) study, high PIVs in patients with cardiovascular diseases were related to worse clinical outcomes, including increased mortality. The study highlighted the use of the PIV as a marker of systemic inflammation and its prognostic value in cardiovascular conditions [16]. In a study by Cetinkaya et al. (2024) on the relationship between the PIV and MACE in patients with CAD, they stated that patients with high PIVs had a significantly higher risk of MACE during follow-up. However, they stated that the PIV is a valuable marker for long-term cardiovascular risk assessment in CAD patients [34]. In the study of Liu et al. (2023), high PIVs were found to be an important determinant of 1-year mortality in heart failure. The study highlighted that higher PIVs were associated with worse functional status and increased systemic inflammation, which are critical factors in the progression and outcomes of heart failure [35]. In our previous study, we demonstrated that the PIV had substantial predictive power, with high values of these indices indicating an elevated risk of both STEMI and mortality [30]. In our study, the PIV was significantly higher in survivors than in non-survivors. ROC curve analysis showed that the PIV had a good discriminatory ability in predicting mortality. Multiple logistic regression analysis confirmed that the PIV was an independent predictor of mortality in PTE patients.

### Limitations of the Study

Our study has some limitations. One limitation is its single-center study nature. Although a detailed examination was carried out, it was achieved by scanning the patients’ files. Another limitation is that we did not include detailed information on the severity of pulmonary embolism (PE), as measured by computed tomography pulmonary angiography (CTPA), nor did we account for D-dimer levels or other relevant clinical markers. Future research should incorporate these parameters to better evaluate the role of TGI and PIV in mortality prediction. Multicenter studies are needed to better determine the prognostic value of the TGI and PIV in PTE patients. However, it should be investigated whether the combination of the TGI and PIV with other parameters increases its prognostic accuracy.

## 5. Conclusions

In conclusion, this study demonstrates a significant association between the TGI, PIV, and mortality in patients with PTE. To our knowledge, this is one of the first studies to comprehensively evaluate these two markers together in a PTE population, highlighting their independent prognostic value. Incorporating these markers into routine clinical assessments may enhance risk stratification and enable more precise identification of high-risk patients, potentially improving management strategies and reducing mortality rates in this population.

The novelty of this study lies in the combined evaluation of metabolic and inflammatory markers, which has not been extensively explored in PTE, despite growing evidence of the role of metabolic and immune-inflammatory pathways in other cardiovascular conditions. Prior studies have largely focused on traditional risk factors, while our findings suggest that the TGI and PIV may serve as easily measurable, clinically relevant predictors of outcomes in PTE. Future research should further explore the mechanisms underlying these associations and evaluate whether targeting these pathways could offer new therapeutic opportunities for improving patient outcomes.

## Figures and Tables

**Table 1:** Comparison of laboratory and socio-demographic findings.

Parameters	Non-Survivors (N = 220) (Mean ± SD/% (*n*))	Survivors (N = 230) (Mean ± SD/% (*n*))	*p*-Value
Age	66.1 ± 11.8	58.3 ± 12.4	0.03
Gender (Male)	55% (121)	45% (104)	0.05
BMI	28.3 ± 5.1	25.7 ± 4.5	0.04
Hypertension	40% (88)	30% (69)	0.04
Diabetes	35% (77)	20% (46)	0.01
Smoking	25% (55)	15% (35)	0.02
Systemic Thrombolytics	35% (77)	50% (115)	0.01
Catheter-Directed Thrombolysis	15% (33)	25% (58)	0.03
EKOS (Endovenous Laser Therapy)	5% (11)	12% (28)	0.04
TGI	12.1 ± 1.5	5.9 ± 1.2	<0.001
PIV	878.2 ± 85.4	254.5 ± 61.1	<0.001

BMI: body mass index, TGI: Triglyceride–Glucose Index, PIV: pan-immune inflammation.

**Table 2:** Univariate logistic regression analysis of factors used for non-survivors.

Variable	Coefficient	Std. Error	z-Value	[95% CI]	*p* Value
const	−3.026	1.512	−2.000	[−6.000, −0.052]	0.045
Age	0.036	0.011	3.273	[0.014, 0.058]	0.001
Gender	0.578	0.321	1.801	[−0.052, 1.208]	0.072
BMI	0.064	0.024	2.678	[0.018, 0.110]	0.007
Hypertension	0.498	0.355	1.404	[−0.197, 1.193]	0.160
Diabetes	0.721	0.392	1.838	[−0.048, 1.490]	0.066
Smoking	0.638	0.387	1.649	[−0.121, 1.397]	0.099
TGI	0.312	0.121	2.587	[0.074, 0.550]	0.010
PIV	0.410	0.180	2.279	[0.058, 0.762]	0.023

BMI: body mass index, TGI: Triglyceride–Glucose Index, PIV: pan-immune inflammation.

**Table 3:** Multiple logistic regression analysis of factors used for non-survivors.

Variable	Coefficient	Std. Error	z-Value	[95% CI]	*p* Value
Const	−3.125	1.478	−2.114	[−6.023, −0.227]	0.034
Age	0.031	0.011	2.818	[0.009, 0.052]	0.005
BMI	0.068	0.022	3.091	[0.025, 0.111]	0.002
TGI	0.297	0.119	2.492	[0.063, 0.531]	0.013
PIV	0.395	0.173	2.282	[0.056, 0.734]	0.022

BMI: body mass index, TGI: Triglyceride–Glucose Index, PIV: pan-immune inflammation.

**Table 4:** ROC analysis results in patients with non-survivors.

Parameter	Cut-Off	Sensitivity	Specificity	AUC (95% CI)	*p* Value
Age	>60	0.60	0.60	0.65 (0.57–0.73)	0.002
BMI	>27	0.65	0.55	0.61 (0.52–0.70)	0.031
TGI	>10	0.78	0.73	0.76 (0.69–0.83)	<0.001
PIV	>500	0.82	0.75	0.79 (0.72–0.86)	<0.001

BMI: body mass index, TGI: Triglyceride–Glucose Index, PIV: pan-immune inflammation.

## Data Availability

The original contributions presented in the study are included in the article, further inquiries can be directed to the corresponding authors.
